# Evaluating the predictive significance of systemic immune-inflammatory index and tumor markers in lung cancer patients with bone metastases

**DOI:** 10.3389/fonc.2023.1338809

**Published:** 2024-01-09

**Authors:** Jinxian He, Gaofeng Liang, Hongyan Yu, Chengbin Lin, Weiyu Shen

**Affiliations:** Department of Thoracic Surgery, Ningbo Medical Center Lihuili Hospital, Ningbo, Zhejiang, China

**Keywords:** lung cancer, bone metastases, predictive modeling, immunotherapy, tumor markers, nomogram

## Abstract

**Objective:**

This study aims to develop a predictive model for identifying lung cancer patients at elevated risk for bone metastases, utilizing the Unified Immunoinflammatory Index and various tumor markers. This model is expected to facilitate timely and effective therapeutic interventions, especially in the context of the growing significance of immunotherapy for lung cancer treatment.

**Methods:**

A retrospective analysis was conducted on 324 lung cancer patients treated between January 2019 and January 2021. After meeting the inclusion criteria, 241 patients were selected, with 56 exhibiting bone metastases. The cohort was divided into a training group (169 patients) and a validation group (72 patients) at a 7:3 ratio. Lasso regression was employed to identify critical variables, followed by logistic regression to construct a Nomogram model for predicting bone metastases. The model’s validity was ascertained through internal and external evaluations using the Concordance Index (C-index) and Receiver Operating Characteristic (ROC) curve.

**Results:**

The study identified several factors influencing bone metastasis in lung cancer, such as the Systemic Immune-Inflammatory Index (SII), Carcinoembryonic Antigen (CEA), Neuron Specific Enolase (NSE), Cyfra21-1, and Neutrophil-to-Lymphocyte Ratio (NLR). These factors were incorporated into the Nomogram model, demonstrating high validation accuracy with C-index scores of 0.936 for internal and 0.924 for external validation.

**Conclusion:**

The research successfully developed an intuitive and accurate Nomogram prediction model utilizing clinical indicators to predict the risk of bone metastases in lung cancer patients. This tool can be instrumental in aiding clinicians in developing personalized treatment plans, thereby optimizing patient outcomes in lung cancer care.

## 1 Introduction

Lung cancer is a highly lethal cancer, causing about one-third of all cancer deaths worldwide (1). This is mainly because early symptoms of lung cancer, such as coughing, are often unremarkable and not quickly alerted (2). Most patients seek medical assistance only when they experience severe symptoms such as hemoptysis and pain. By this time, the cancer has already progressed to an advanced stage or is detected by physical examination in the absence of apparent symptoms. However, with advances in various types of treatments, such as targeted therapies and immunotherapies, the death rate from lung cancer is decreasing every year. According to the U.S. Cancer Data 2021 (3), the mortality rate of lung cancer decreased by nearly half between 2014 and 2018, doubling the rate of decline, which is closely related to the reduction of smoking and the improvement of early diagnosis and treatment outcomes. The incidence of lung cancer is relatively low before the age of 50 years, but the risk increases progressively with age. Low-dose spiral CT, lung cancer screening, is recommended for high-risk groups who are older, long-term smokers, and exposed to occupational pollution; it is significantly more effective than ordinary chest radiographs and can reduce lung cancer mortality by 20%, which is essential for early detection of lung cancer (4).

Distant metastasis often occurs when lung cancer progresses in the course of the disease, and common sites of metastasis include intracranial, bone, lymph nodes, and so on (5). Among them, bones, especially load-bearing bones such as the middle shaft bone, are common remote metastatic sites of lung cancer (6). Once the bones are eroded by tumor cells, in addition to possible pathological fractures, they may also lead to the emergence of bone-related problems such as hypercalcemia, spinal cord injury, and pain, which negatively affect the quality of life of patients (7). At the same time, this further exacerbates the financial pressure on cancer patients as the treatment of pain and pathological fractures requires operations such as surgery and radiotherapy (8). This also means it is crucial to search for and identify risk factors for bone metastasis in lung cancer to detect and predict bone metastasis promptly.

Recently, many researchers have begun to focus on the factors associated with predicting bone metastasis in lung cancer and have attempted to construct predictive models (9). Previous studies have identified factors such as blood calcium, T4 stage, N3 stage, p-III stage, non-squamous cell carcinoma, bone salivary protein BSP expression, elevated carcinoembryonic antigen levels, and high alkaline phosphatase as risk factors for bone metastasis in lung cancer (10, 11). However, there is a relative lack of studies on the relationship between inflammatory response and lung cancer bone metastasis. Inflammatory response is essential in the tumor microenvironment and is closely related to tumor generation, development, aggression, and metastasis (12). The systemic immune-inflammatory index (SII) is a novel prognostic predictor calculated by multiplying platelets by the absolute value of neutrophils and dividing by the total value of lymphocytes (13). The formation of new blood vessels is one of the essential conditions for further tumor progression and distant metastasis. Therefore, circulating vascular endothelial growth factor (VEGF) levels are gaining wider acceptance as a prognostic factor in cancer patients’ diagnostic and therapeutic evaluation (14).

Nevertheless, the value of the systemic immunoinflammatory index (SII) in predicting bone metastasis in lung cancer is unclear. Bone metastasis is a standard process in which primary cancers undergo metastasis. When bone metastasis occurs in lung cancer, it not only aggravates the patient’s condition but also reduces the patient’s survival rate. For lung cancer patients suspected of having bone metastases, ECT (single photon emission computed tomography) and PET (positron emission tomography) are two commonly used diagnostic imaging methods that can effectively detect and localize cancer cells in the bones (15, 16). However, these tests involve radioactive substances, and prolonged or frequent exposure may pose certain health risks for patients and medical personnel.

Therefore, if other biological indicators or risk factors can predict the possibility of bone metastasis of lung cancer, it will be possible to select patients who need to undergo radiological examinations more accurately, thus reducing the risk of unnecessary radiation exposure. This will not only help protect the health of patients and medical staff but also save medical resources and improve the efficiency of diagnosis.

## 2 Methodology and information

### 2.1 Sample collection and ethical approval

**Figure 1:**
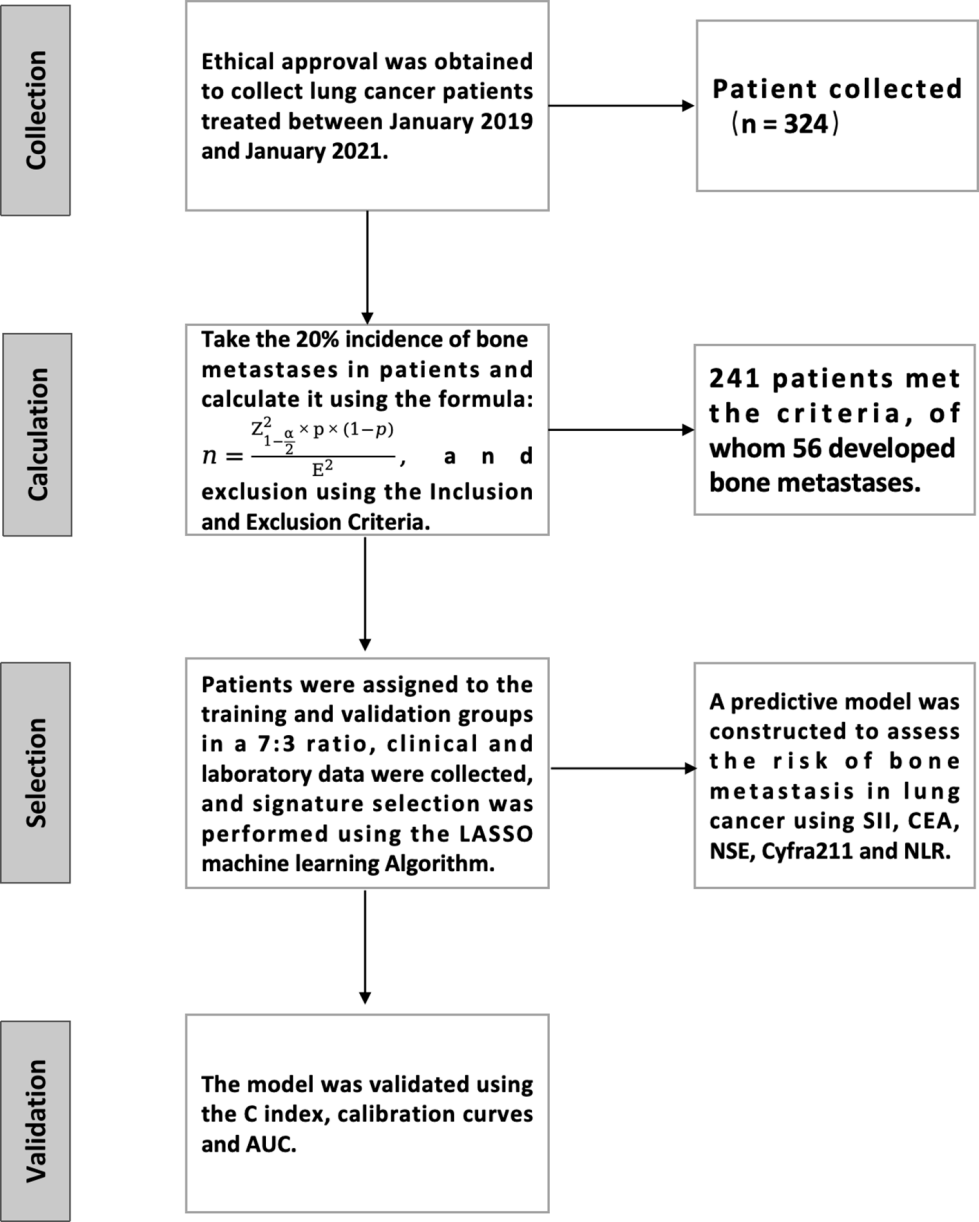
Flow chart of sample inclusion and exclusion.

The process of this study is detailed in **Figure 1**. A retrospective analysis was conducted on 324 lung cancer patients treated at our hospital from January 2019 to January 2021. The study has been approved and endorsed by the Medical Ethics Committee of Li Huili Hospital, Ningbo Medical Centre, with the approval number Li Huili Hospital Ethical Approval 2023 Study No. 233. Acceptance number: KY2023SL233-01.

### 2.2 Sample size calculation

Based on the available risk queries, we found that the incidence of bone metastases ranged from 15% to 25% (10). We took the 20% incidence rate and used the following formula: $n=\frac{Z_{1-\frac{\alpha }{2}}^{2}\times p\times (1-p)}{E^{2}}$ ,: Whetwoe ${\text{Z}}_{1-\frac{\text{α}}{2}}$ is taken as 1.96, p is the prevalence of 20%, E is the maximum error taken as 0.05, and the final calculation is that 246 patients are needed.

### 2.3 Inclusion and exclusion criteria

Inclusion criteria comprised: (1) newly diagnosed malignant lung tumor patients; (2) patients presenting initially to our hospital without prior antitumor treatment (surgery, radiotherapy, chemotherapy, immunotherapy, or targeting); (3) availability of complete patient data; (4) confirmed diagnosis of lung cancer bone metastasis, either by clinical/pathological diagnosis and bone lesion biopsy or by typical imaging manifestations (17); (5) clinical TNM (cTNM) stage ≥ II.

Exclusion criteria included: (1) incomplete medical records; (2) significant comorbidities; (3) pre-existing bone-related diseases; (4) serious infections; (5) prior diagnosis or antitumor therapy in another hospital; (6) imaging suggestive of bone destruction but lacking comprehensive bone imaging.

### 2.4 Sample selection

Of the initial 324 patients, 241 met the inclusion criteria. Of these patients, 56 (23.23%) had bone metastases. For the study, we divided these patients into a training group (169) and a validation group (72) with an approximate ratio of 7:3. During the grouping process, we used a RAND function to assign patients randomly. Specifically, we generated a random number for each eligible patient. Patients were then transferred to the training and validation groups in a 7:3 ratio based on the order of these numbers. This method ensured the groupings’ randomization and helped us reduce potential selection bias, making the study results more reliable and valid.

### 2.5 Data collection

Data were retrieved from electronic medical records and outpatient review documents. Collected data included demographics (age, Gender, smoking status, BMI), pathological staging, and laboratory data (neutrophil count, lymphocyte count, peripheral platelet count, CEA, Cyfra21-1, NSE levels). The following calculations were made:


$$\begin{array}{c} Systemic\;Immunoinflammatory\;Index\;(SII)\\ =(platelet\;count\times neutrophil\;count)/lymphocyte\;count. \end{array}$$

$$\begin{array}{c} Neutrophil\;to\;Lymphocyte\;Ratio\;(NLR)\\ =neutrophil\;count/lymphocyte\;count. \end{array}$$

### 2.6 Signature selection steps

Data from the 241 patients were analyzed using SPSS 26.0. The training group data underwent feature selection using the LASSO machine learning algorithm.

### 2.7 Internal validation process

Model validation involved the C-index, calibration curve, and ROC curve area under the curve (AUC). The C-index assessed concordance between predicted outcomes and actual observations. The calibration curve evaluated the fit between anticipated and observed risks. Decision curve analysis determined the clinical benefits, aiding in identifying high-risk patients for intervention and sparing low-risk patients from unnecessary treatments.

### 2.8 Statistical analysis

Statistical analysis utilized SPSS 26.0 and R software. The “glmnet” package was employed for the LASSO model construction, “rms” for plotting column line graphs and determining the C-index, and “rocr” for ROC analysis. A P value< 0.05 was considered statistically significant.

## 3 Result

### 3.1 Screening of LASSO signature variables

**Figure 2:**
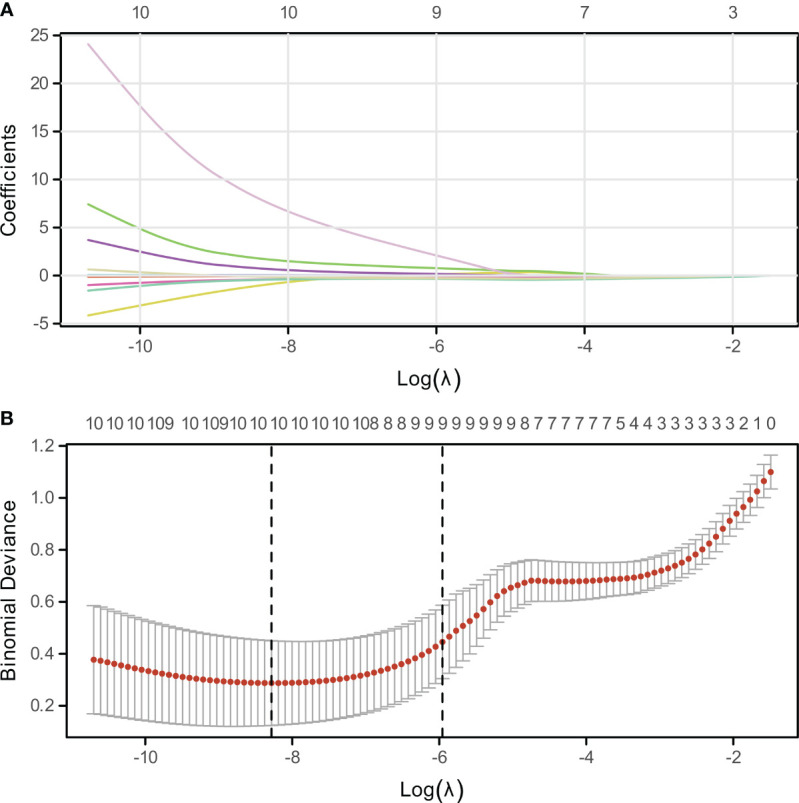
Variable Selection via LASSO Regression. **(A)** Visualization of non-zero coefficient genes utilized in model construction, indicating their relative importance. **(B)** Plot of log-lambda values against cross-validation error, highlighting the optimal lambda value corresponding to the most predictive subset of genes.

The LASSO algorithm and 10-fold cross-validation were utilized to identify significant variables associated with bone metastasis in lung cancer. The optimal value of the tuning parameter lambda.1se was determined to be 0.0025887, as illustrated in **Figures 2A, B**. Through this rigorous selection process, nine key variables were identified: age, Gender, tumor type, smoking history, SII, CEA, NSE, Cyfra211, and NLR, as depicted in **Figure 2**.

### 3.2 Univariate analysis of characteristic variables

First, we assigned values for age, gender, tumor type, smoking history, SII, CEA, NSE, Cyfra211, and NLR (**Table 1**). Subsequently, by univariate analysis, we found that SII, CEA, NSE, Cyfra211, and NLR were strongly associated with bone metastasis in lung cancer patients (P< 0.0001, **Table 2**). In addition, we also compared the differences in SII, CEA, NSE, Cyfra211, and NLR between patients in the modeling and validation groups. The results showed no statistical difference in SII, CEA, NSE, Cyfra211, and NLR between patients in the modeling and the validation groups (P > 0.05, **Table 3**).

**Table 1:** Table of Assignments.

Variables	Assign a value to something
**Age**	≥60 years = 0,<60 years = 1
**Gender**	Male = 0, female = 1
**BMI**	≥25kg/m^2 =^ 0,<25kg/m^2 =^ 1
**Tumor type**	Squamous carcinoma = 0, adenocarcinoma = 1, other = 2
**Smoking history**	Present = 0, absent = 1
**SII**	≥490 = 0,<490 = 1
**CEA(ng/mL)**	≥6.34 = 0,<6.34 = 1
**NSE(ng/mL)**	≥15.65 = 0,<15.65 = 1
**CYFRA21-1(ng/mL)**	≥5.26 = 0,<5.26 = 1
**NLR**	≥3.25 = 0,<3.25 = 1
**Bone metastasis**	Present = 0, absent = 1

Body Mass Index (BMI), CEA (Carcinoembryonic Antigen), Cyfra21-1 (Cytokeratin 19 Fragment 21-1), NSE (Neuron Specific Enolase), Systemic Immunoinflammatory Index (SII), Neutrophil to Lymphocyte Ratio (NLR).

**Table 2:** Univariate analysis of variance.

Variables		Bone metastasi s(n=40)	No bone metastases (n=129)	χ2-value	P-value
**Age**				0.135	0.712
	≥60 years	19	57		
	<60 years	21	72		
**Gender**				2.955	0.085
	Male	31	81		
	Female	9	48		
**Tumor type**				0.714	0.699
	Squamous carcinoma	20	61		
	Adenocarcinoma	17	62		
	Other	3	6		
**Smoking history**				0.340	0.559
	Yes	31	94		
	No	9	35		
**SII**				35.031	<0.0001
	≥490	34	41		
	<490	6	88		
**CEA(ng/mL)**				34.980	<0.0001
	≥6.34	23	16		
	<6.34	17	113		
**NSE(ng/mL)**				44.363	<0.0001
	≥15.65	33	31		
	<15.65	7	98		
**CYFRA21-1(ng/mL)**				16.198	<0.0001
	≥5.26	27	41		
	<5.26	13	88		
**NLR**				25.329	<0.0001
	≥3.25	30	39		
	<3.25	10	90		

CEA, carcinoembryonic antigen; Cyfra21-1, Cytokeratin 19 Fragment 21-1; NSE, neuron-specific enolase; SII, Systemic Immunoinflammatory Index; NLR, Neutrophil to Lymphocyte Ratio.

**Table 3:** Comparison of clinical data between patients in the training group and the validation group.

Considerations		Validation group (n=72)	Training group (n=169)	χ2-value	P-value
**SII**				0.346	0.556
	≥490	29	75		
	<490	43	94		
**CEA (ng/mL)**				0.008	0.928
	≥6.34	17	39		
	<6.34	55	130		
**NSE (ng/mL)**				1.142	0.285
	≥15.65	33	64		
	<15.65	40	105		
**CYFRA21-1 (ng/mL)**				0.649	0.420
	≥5.26	33	68		
	<5.26	39	101		
**NLR**				0.470	0.492
	≥3.25	26	69		
	<3.25	46	100		

CEA, carcinoembryonic antigen; Cyfra21-1, Cytokeratin 19 Fragment 21-1; NSE, neuron-specific enolase; SII, Systemic Immunoinflammatory Index; NLR, Neutrophil to Lymphocyte Ratio.

### 3.3 Training the risk prediction model for bone metastasis in lung cancer

**Figure 3:**
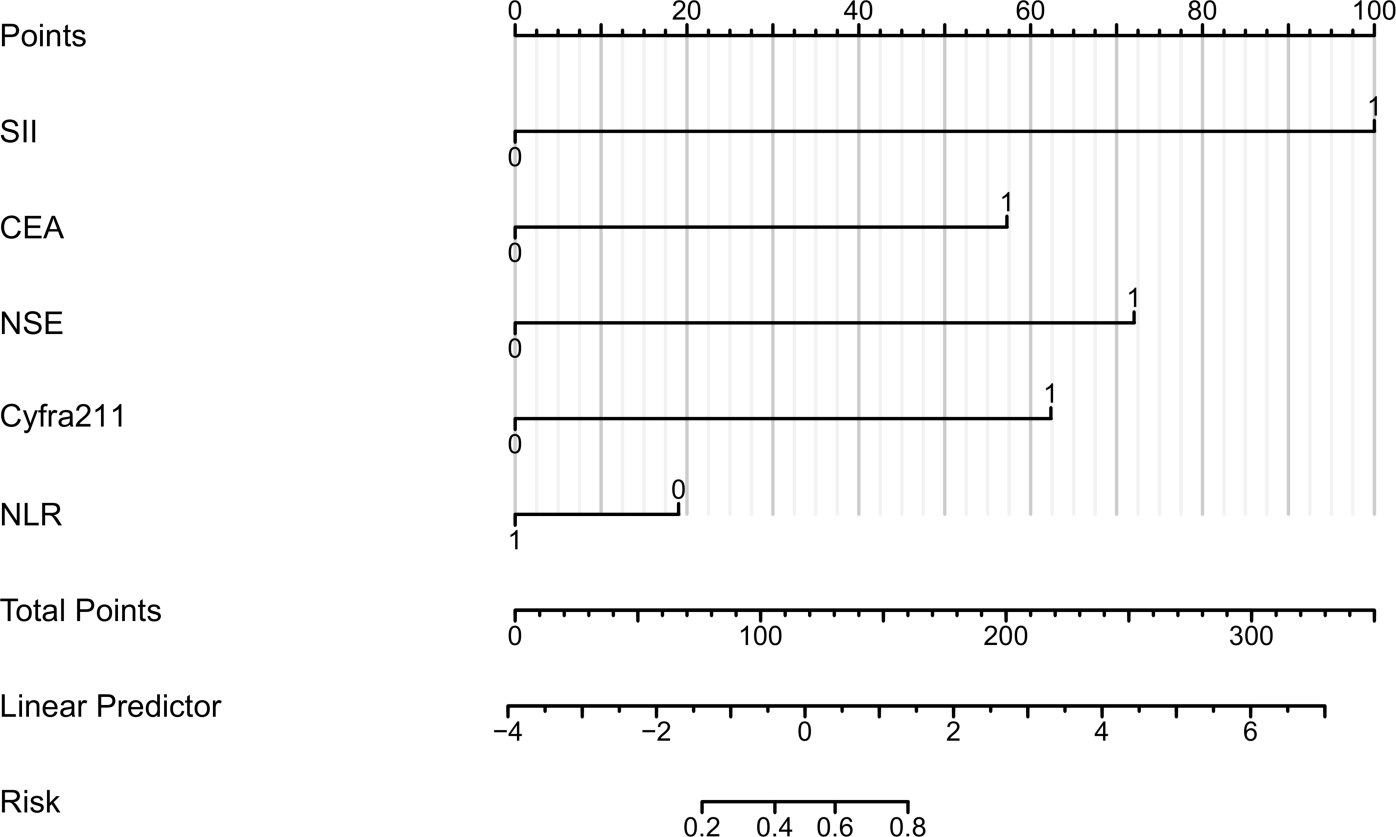
Columnar Representation of Risk Factors for Bone Metastasis in Lung Cancer. This figure presents a column chart indicating the scoring of variables, where CEA is carcinoembryonic antigen, Cyfra21-1 represents cytokeratin 19 fragment 21-1, NSE denotes neuron-specific enolase, SII is the systemic immunoinflammatory index, and NLR stands for the neutrophil to lymphocyte ratio. The aggregate score correlates with the risk of bone metastasis.

For the five characteristic variables screened by univariate screening, a column chart model was constructed to predict the risk of bone metastasis in lung cancer (**Figure 3**). In the visualization of the risk prediction column chart, Points represent the corresponding scores of the variables, and different values of the variables correspond to varying values of Points. The TotalPoints are obtained by summing up the scores of each variable. By analogy, the risk of lung cancer bone metastasis corresponding to the total points of each patient can be read out from the Risk of Lung Cancer Bone Metastasis in the lower part of the graph, which is helpful for individualized prediction of lung cancer bone metastasis in clinical practice.

### 3.4 Risk prediction and model validation for lung cancer bone metastasis

**Figure 4:**
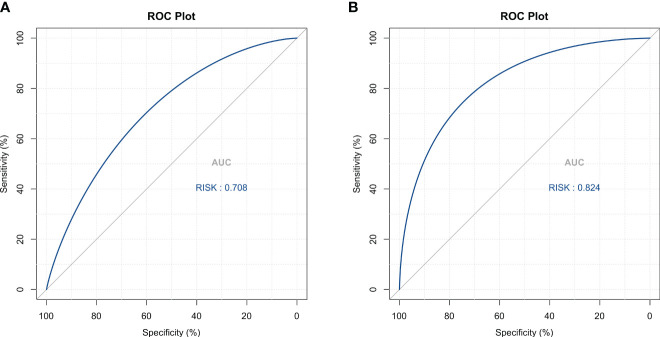
Discriminative Analysis Using ROC Curves. **(A)** ROC curve analysis for the training cohort, demonstrating the model’s capacity to distinguish between lung cancer cases with and without bone metastasis. **(B)** ROC curve validation for the external cohort, confirming the model’s discriminative performance.

**Figure 5:**
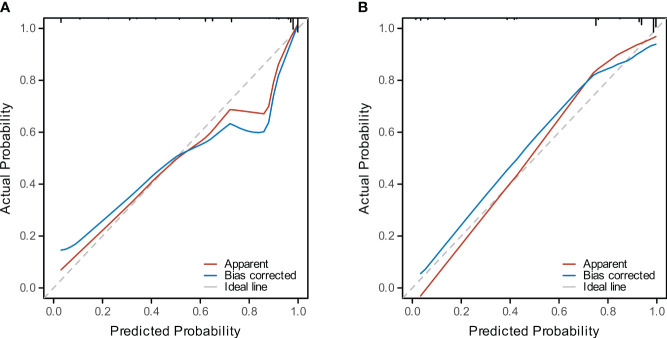
Calibration of the Predictive Model. **(A)** The calibration curve for the training cohort model depicts the concordance between predicted and observed bone metastasis in lung cancer. **(B)** The calibration curve for the validation cohort illustrates the model’s predictive accuracy.

**Figure 6:**
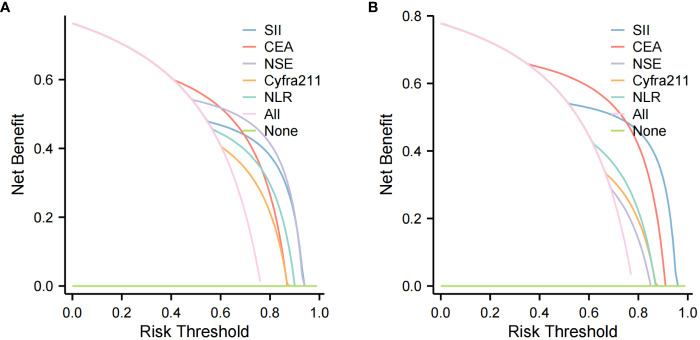
Clinical Utility Assessment with Decision Curve Analysis (DCA). **(A)** DCA for the training cohort, assessing the predictive model’s clinical benefit in diagnosing lung cancer bone metastasis. **(B)** DCA for the validation cohort, evaluating the model’s net benefit across various decision thresholds.

Four methods of internal and external validation of the model, including the ROC curve, C-index, and calibration curve, were used to obtain the validity of the risk prediction model: (1) The AUC of internal validation was 0.708, and the AUC of external validation was 0.824, which indicated that the prediction model had an excellent discriminatory ability (**Figures 4A, B**). (2) The calibration curves of internal validation and external validation showed that the predicted probability of bone metastasis of lung cancer matched well with the actual situation, indicating the accuracy of the prediction model (**Figures 5A, B**); (3) The C-indexes of the internal validation and the external validation were C-index: 0.936 (0.897 - 0.975) and C-index: 0.924 (0.842 - 1.007), indicating that the actual probability of bone metastasis of lung cancer had good discriminative ability (**Figures 4A, B**). that the actual probability of bone metastasis in lung cancer is in good agreement with the predicted probability. (4) The DCA curves of internal and external validation showed that the predictive model showed good clinical net gain under different threshold probabilities when predicting the probability of DR, confirming its practicality (**Figures 6A, B**).

## 4 Discussion

In our study of 241 lung cancer patients, we explored risk factors that promote bone metastasis and developed a diagnostic model. Our findings are consistent with previous studies showing that gender, age, and smoking habits do not have a significant effect on the likelihood of bone metastasis. Although some studies have shown that smokers are more likely to develop bone-related complications (18), the role of age in bone metastasis remains controversial. For example, Wang et al. (19) reported an increase in bone metastases in lung cancer patients over 55 years of age. At the same time, another study (20) found a higher incidence of bone metastases among younger patients. However, our study did not find a direct correlation between age and bone metastasis, which suggests that further investigation of this relationship is needed.

The inflammatory response, a key player in the tumor microenvironment, is intricately linked with tumor initiation, progression, invasion, and metastasis (21). Long-term exposure to exogenous inflammatory factors can increase cancer risk and progression (22, 23). The Neutrophil-to-Lymphocyte Ratio (NLR), an indicator of systemic inflammation, has been linked to poor prognosis in cancer (24, 25). For instance, Sun et al. (26) found a high NLR associated with poorer overall survival (OS) and progression-free survival (PFS) in advanced NSCLC. In addition, several studies have found that high levels of NLR are positively associated with poor prognosis in lung cancer (27, 28). Our study corroborates these findings, with NLR emerging as an independent risk factor for bone metastasis in lung cancer. In the current study, NLR was significantly higher in both bone metastasis groups compared with non-bone metastasis groups, and regression analysis showed that it was an independent risk factor for bone metastasis. This suggests that NLR is highly valuable in predicting and diagnosing bone metastasis in lung cancer patients.

Similarly, the Systemic Immune-Inflammatory Index (SII) has been recognized as a prognostic factor in various solid tumors (29, 30). These studies have shown that patients with high levels of SII are more likely to develop bone metastases and have a higher proportion of T-stage and lymph node metastases. Our findings suggest that elevated SII levels are indicative of a higher likelihood of bone metastases in lung cancer patients.

CEA, a glycoprotein crucial in cell adhesion, is usually only produced during fetal life. Many studies have shown that elevated CEA is strongly associated with the development of colorectal cancer (31). In addition, NSE, a cell-specific isoenzyme, usually is present only in specific tissues. Still, during malignant tumor proliferation, the level of NSE in body fluids is increased, which is valuable for diagnosing, staging, and treating related neuroendocrine tumors (32). Cyfra21-1 is a cytokeratin expressed in simple epithelia, including bronchial epithelium, and in malignant tumors that develop from these cells (33). As a serum marker for lung cancer, Cyfra21-1 is commonly used for lung cancer screening, treatment, and efficacy monitoring, and Okamura et al. (34) found that both CEA and Cyfra21-1 had good sensitivity and specificity for diagnosing lung cancer in a high-risk-population. In lung cancer, elevated levels of these markers are associated with bone metastasis. For example, elevated NSE levels correlate with the number of lung cancer bone metastases (35), while high Cyfra 21-1 levels are linked to distant metastasis (36). Therefore, these findings suggest that lung cancer tumor markers are closely related to bone metastasis of lung cancer. Changes in lung cancer tumor markers should be paid attention to in the process of cancer diagnosis and treatment, significantly when Cyfra 21-1, NSE, and CEA are elevated simultaneously; timely attention should be paid to whether there is the occurrence of bone metastasis.

This study successfully developed a model to predict bone metastases in lung cancer, aiding in determining the appropriateness of immunotherapy. High-risk patients may benefit from early immunotherapy to prevent or delay bone metastasis, while low-risk patients might avoid premature treatment. This model lays the groundwork for personalized immunotherapy regimens. However, there are limitations to our study. Being a single-center study, the generalizability of our findings needs further validation with broader data sets. Additionally, the model was validated only using data from our center, necessitating external validation to minimize selection bias. Besides, future research will focus on homogeneously treated patients to study PFS and incorporate more sophisticated machine learning or subgroup analysis methods to refine the predictive model. This will allow for a more effective clinical assessment of lung cancer patients at risk of bone metastasis.

## 5 Conclusion

This study successfully developed and validated an innovative, objective, and accurate nomogram prediction model for predicting the risk of bone metastasis in lung cancer to show high accuracy. The model provides clinicians with a valuable tool for risk assessment and personalized treatment planning. With early immunotherapy, high-risk patients may benefit from preventing or delaying bone metastases, while low-risk patients may avoid premature treatment. This model lays the foundation for personalized immunotherapy regimens.

## Data availability statement

The datasets presented in this study can be found in online repositories. The names of the repository/repositories and accession number(s) can be found in the article/supplementary material. The datasets generated and analyzed during the current study are available in the Figshare repository. These can be accessed through the following DOI: 10.6084/m9.figshare.24564493.

## Ethics statement

The studies involving humans were approved by the Medical Ethics Committee of Li Huili Hospital, Ningbo Medical Centre (No. KY2023SL233-01). The studies were conducted in accordance with the local legislation and institutional requirements. The ethics committee/institutional review board waived the requirement of written informed consent for participation from the participants or the participants’ legal guardians/next of kin because Retrospective studies do not require informed consent.

## Author contributions

JH: Conceptualization, Data curation, Investigation, Writing – original draft. GL: Formal analysis, Methodology, Writing – original draft. HY: Validation, Visualization, Writing – original draft. CL: Project administration, Validation, Writing – original draft. WS: Conceptualization, Supervision, Writing – review & editing.
